# Myocardial fibrosis quantified by the extracellular extravascular volume fraction is associated with the left ventricular sphericity index and the left atrial volume index

**DOI:** 10.1186/1532-429X-14-S1-P294

**Published:** 2012-02-01

**Authors:** Diego Moguillansky, Timothy C Wong, Christopher G Meier, Stephen M Testa, David Testa, William J Ceyrolles, Kayla Piehler, Peter Kellman, Erik B Schelbert

**Affiliations:** 1 Cardiovascular Disease, University of Pittsburgh Medical Center, Pittsburgh, PA, USA; 2 Congenital Heart Center, University of Florida, Gainesville, FL, USA; 3 Laboratory of Cardiac Energetics, National Institutes of Health/NHLBI, Bethesda, MD, USA

## Summary

The objective of this study is to test the hypothesis that quantitative measures of myocardial fibrosis such as the myocardial extravascular extracellular volume fraction (Ve) are associated with markers of adverse cardiac remodeling such as the left atrial volume index (LAVi) and left ventricular sphericity index (Si).

## Background

LAVi and Si are intermediate phenotypes that precede adverse outcomes. Myocardial fibrosis is quantifiable with contemporary CMR techniques, is treatable, and may represent a therapeutic target when these intermediate phenotypes are present.

## Methods

We measured myocardial Ve in 267 individuals referred for CMR without confounders such as myocardial infarction, where Ve= [λ ρ (1-Hct) - Vp]; the specific density of myocardial tissue, ρ=1.05; the myocardial plasma volume fraction, Vp=0.045, and λ=ΔR1myocardium/ ΔR1blood. T1 was measured with an ECG-gated MOLLI sequence acquired before and 20 minutes after a gadolinium contrast bolus (0.2 mmol/kg). LAVi and Si were measured from standard SSFP cine images: LAVi =[8/3π [(A1)(A2)/L] where A1 and A2 are LA areas from end-systolic 2-chamber and 4-chamber views, L represents the shortest anteroposterior LA dimension from either view, and Si =EDV/[LAX^3 π /6), where EDV is the end-diastolic volume measured from short axis stacks and LAX^3 is the cube of the long axis diastolic dimension from a 4 chamber view. Multivariable linear regression models quantified the association of Ve with LAVi and Si, adjusting for key characteristics identified by stepwise selection.

## Results

Ve was associated with LAVi (t value 4.5, p<0.001), and this association remained after adjusting for age, ejection fraction, EDV index, and left ventricular mass index (t value 2.8, p=0.005). Ve was also associated with Si (t value 5.6, p<0.001), and this association remained after adjusting for age, gender, EDV index, body mass index, and left ventricular mass index (t value 2.18, p=0.036).

## Conclusions

Ve is associated with key intermediate phenotypes that indicate adverse cardiac remodeling such as LAVi and Si.

## Funding

Dr Erik Schelbert is supported by a University of Pittsburgh Foundation Grant, and by an American Heart Association Scientist Development Grant (09SDG2180083).

